# Detection of *Arcobacter* species in different intestinal compartments of broiler chicken during slaughter and processing

**DOI:** 10.1002/mbo3.1106

**Published:** 2020-08-24

**Authors:** Antje Schönknecht, Thomas Alter, Greta Gölz

**Affiliations:** ^1^ Institute of Food Safety and Food Hygiene Freie Universität Berlin Berlin Germany

## Abstract

*Arcobacter* spp. are commonly present on meat products. However, the source of contamination on chicken meat is under dispute. Since different studies reported contradictory results on the occurrence of *Arcobacter* spp. inside the intestinal tract of chicken, our study examined four intestinal compartments at four significant production steps during broiler slaughter and processing in the slaughterhouse. Altogether, 157 intestinal tracts from 19 flocks were examined qualitatively and semiquantitatively applying a selective enrichment. Further verification was performed by mPCR and *rpoB* sequencing. *Arcobacter* spp. were only detected sporadically in intestinal contents after bleeding (2/32) and in none after scalding (0/32). After defeathering, *Arcobacter* spp. were detected in 62% (18/29) of the intestinal contents with 28% (8/29) of the duodenal, 21% (6/29) of the jejunal, 3% (1/29) of the cecal, and 55% (16/29) of the colonic samples tested positive with loads up to 24,000 MPN/g in the colonic content. Further 88% (7/8) of colonic tissue samples were tested positive. After evisceration, the prevalences (58/64) and loads of *Arcobacter* spp. display comparable levels in the intestinal contents like after defeathering. In conclusion, our data point out that *Arcobacter* spp. are most likely detected in the colonic intestinal compartment of the chicken after defeathering and evisceration. Therefore, not only cross‐contamination originating from the environment inside the slaughterhouse may cause carcass contamination with *Arcobacter* spp. on broiler chicken carcasses. The detection of *Arcobacter* spp. in duodenal and jejunal contents as well as in the colonic tissue indicates that there possibly exists an *Arcobacter* reservoir inside the chicken.

## Graphical abstract

*Arcobacter* spp. are commonly present on chicken meat products, and since cross‐contamination most probably takes places in the chicken slaughterhouse, we examined 157 intestinal tracts from 19 flocks at four significant production steps during broiler slaughter and processing in the slaughterhouse. Our data point out that *Arcobacter* spp. are most likely detected in the colonic intestinal compartment of the chicken after defeathering and evisceration, but the detection of *Arcobacter* spp. in duodenal and jejunal contents as well as in the colonic tissue indicates that not only cross‐contamination originating from the environment inside the slaughterhouse may cause contamination with *Arcobacter* spp. on chicken carcasses.

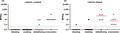

## 1 INTRODUCTION

*Arcobacter* belong to the family of *Campylobacteraceae*. In contrast to *Campylobacter*,* Arcobacter* spp. are aerotolerant and psychrophilic. Three species, namely *A*.* butzleri*,* A*.* cryaerophilus*, and *A*.* skirrowii* are thought to be associated with clinical symptoms in animals. Cases of diarrhea, enteritis, and abortion have been reported in pigs, cattle, and sheep (Ho, Lipman, & Gaastra, 2006; On, Jensen, Bille‐Hansen, Jorsal, & Vandamme, 2002; Vandamme et al., 1992), as well as mastitis in cattle (Logan, Neill, & Mackie, 1982; Vandamme et al., 1992). However, most authors regard *Arcobacter* spp. in animals as commensals (Ramees et al., 2017). Over the last years, attempts were made to assess the impact of *Arcobacter* on humans. In 2002, the International Commission on Microbiological Specifications for Foods (ICMSF) classified the species *A*.* butzleri* and *A*.* cryaerophilus* as a serious hazard for human health (ICMSF, 2002). Several sporadic cases of gastroenteritis, bacteremia, endocarditis, and peritonitis associated with *Arcobacter* have been reported in humans (Ho et al., 2006). Furthermore, a large study in Belgium determined *Arcobacter* as the fourth most common pathogen group in fecal samples of enteritis patients (Van den Abeele, Vogelaers, Van Hende, & Houf, 2014).

*Arcobacter* spp. were isolated from various sources like feces, sewage, water, seafood, milk, vegetables, and meat products (Collado & Figueras, 2011; Ramees et al., 2017; Wesley & Miller, 2010). The prevalence of *Arcobacter* spp. in meat products is high, especially in products of poultry origin, followed by pork and beef (Ho et al., 2006; Kabeya et al., 2004). The contamination of poultry meat products most probably occurs during processing in the poultry slaughterhouse (Gude, Hillman, Helps, Allen, & Corry, 2005; Hsu & Lee, 2015). While *Arcobacter* spp. were detected in the intestinal content of chicken in several studies (Ho, Lipman, & Gaastra, 2008; Van Driessche & Houf, 2007), others detected them only on chicken carcasses and in the environment inside the slaughterhouse (Atabay & Corry, 1997; Gude et al., 2005; Houf, De Zutter, Van Hoof, & Vandamme, 2002). Since there is no standardized protocol for the detection of *Arcobacter* spp., various methods have been applied in corresponding studies, which makes it difficult to compare results of different studies.

However, since *Arcobacter* spp. are commonly present on poultry products (Houf et al., 2002) and may pose a hazard to human health, it is necessary to clarify the routes of transmission of *Arcobacter* spp. in the chicken processing chain. The purpose of this study was to examine four sections of the intestinal tract (duodenum, jejunum, cecum, colon) of broiler chicken at four significant production steps (bleeding, scalding, defeathering, evisceration) along the slaughter line on a qualitative and semiquantitative level.

## 2 MATERIALS AND METHODS

### 2.1 Sample collection and further processing

All samples were obtained from one slaughterhouse on 13 non‐consecutive days over 20 months. Whole carcasses from a total of 19 flocks were collected at different stages along the process chain. For each flock, samples were collected at different processing points. Samples of at least 7 different flocks were collected at 4 different days for each processing point. In total, 32 carcasses after bleeding, 32 after scalding, and 29 after defeathering were investigated. For all flocks included in the study the already separated intestines after evisceration (*n* = 64) were also collected. Additionally, 4 carcasses of two different flocks were collected at each of the four sampling points (*n* = 32) to compare the *Arcobacter* spp. load in colonic tissue versus content. Furthermore, environmental samples were taken in the slaughterhouse, including scalding water (*n* = 6) and plucking fingers (*n* = 6), which were dismantled from the defeathering machine to be examined. All samples were stored at 4°C until further processing within 24 hr. After removing the intestinal tracts from the carcasses, the intestinal content of duodenum (pars descendens duodeni and pars ascendens duodeni), jejunum (without the distal part of the ileum), both caeca, and colon were aseptically collected. Concerning the samples where colonic tissue and colonic content were analyzed in parallel, the colonic section was opened longitudinally after collecting the content and rinsed with aseptic water. The tissue with the intestinal mucosa was incised and homogenized in *Arcobacter* enrichment broth.

### 2.2 Isolation and verification of *Arcobacter* spp.

All incubation steps were performed at 30°C for 48 hr under microaerobic conditions. For qualitative and semiquantitative detection, 1 g of each sample was added to 9 ml *Arcobacter* enrichment broth containing 24 g/L *Arcobacter* broth (Oxoid), selective supplement: 100 mg/L 5′‐fluorouracil, 10 mg/L amphotericin B, 16 mg/L cefoperazone, 32 mg/L novobiocin, 64 mg/L trimethoprim (Sigma‐Aldrich), and 5% lysed horse blood (Oxoid), according to Houf, Devriese, De Zutter, Van Hoof, and Vandamme (2001). The samples were homogenized for 2 min with a stomacher blender.

To process the scalding water, 50 ml was centrifuged for 10 min at 5000 × *g*. The supernatant was discarded, and 45 ml of *Arcobacter* enrichment broth was added to the remaining sediment of 5 ml and thoroughly mixed for 2 min. The plucking fingers were processed by adding them to a tube containing 10 ml *Arcobacter* enrichment broth, mixing them thoroughly for 2 min. The remaining liquid was incubated as described above.

For semiquantitative detection, serial 10‐fold dilutions of the initial dilutions were prepared in *Arcobacter* enrichment broth. After incubation, 10 µl of each dilution was transferred to *Arcobacter* selective agar plates composed of *Arcobacter* enrichment broth (without lysed horse blood) and 1.2% Agar Bacteriological No. 1 (Oxoid) and further incubated.

For the qualitative detection of *Arcobacter* spp., the homogenates were incubated as described above before plating 50 µl on *Arcobacter* selective agar plates. Suspicious colonies (diameter of 1 mm, beige to transparent) were picked and subcultured on Mueller‐Hinton agar plates (Oxoid) supplemented with 5% defibrinated sheep blood (MHB) and incubated. To confirm the presence of *Arcobacter* spp., DNA was isolated from any dilution that showed bacterial growth on the semiquantitative plates and of suspicious colonies grown on MHB plates from the qualitative samples using the Chelex method as described previously (Karadas et al., 2013) and subsequently identified by mPCR according to Houf, Tutenel, De Zutter, Van Hoof, and Vandamme (2000). In brief, the total volume of 25 µl PCR reaction mixture included 2 µl DNA template, 2.5 µl of 10× PCR buffer (Qiagen), 2.8 mM MgCl_2_, 0.2 mM of each dNTP (Thermo Fisher Scientific), 0.75 U of *Taq* DNA polymerase (Qiagen), 1 µM of each primer ARCO, BUTZ, CRY1, CRY2, and 0.5 µM of the primer SKIR (Primers listed in Table 1). After an initial denaturation step (94°C for 2 min), the PCR involved 32 cycles of denaturation (94°C for 45 s), primer annealing (61°C for 45 s), chain extension (72°C for 30 s), and a final elongation step (72°C for 5 min). After gel electrophoresis in 3% agarose gel, PCR products were visualized with GR Green (Excellgen) under UV light.

**TABLE 1:** Primers used in this study

Primer	Sequence	Amplicon	References
ARCO R	CGTATTCACCGTAGCATAGC		Houf et al. (2000)
BUTZ F	CCTGGACTTGACATAGTAAGAATGA	401	
SKIR F	GGCGATTTACTGGAACACA	641	
CRY 1	TGCTGGAGCGGATAGAAGTA	257	
CRY2	AACAACCTACGTCCTTCGAC		
CamRpoB‐L	CCAATTTATGGATCAAAC	524	Korczak et al. (2006)
RpoB‐R	GTTGCATGTTNGNACCCAT		

The semiquantitative load of *Arcobacter* spp. was determined according to the MPN‐method based on ISO/TS10272‐3:2010/Cor.1:2011 for detection and quantification of *Campylobacter* spp., by adjusting media, incubation time and temperature, and by reducing the sample weight to 1 g. The confirmation of the presence of *Arcobacter* spp. was verified by the mPCR above mentioned (Houf et al., 2000).

For all qualitatively detected isolates identified as *Arcobacter* spp. by mPCR, species verification was performed by *rpoB* sequencing according to (Korczak et al., 2006). In brief, the total volume of 50 µl PCR reaction mixture included 4 µl DNA template, 5 µl 10× PCR buffer, 2.5 mM MgCl_2_, 0.2 mM of each dNTP, 1 U of *Taq* DNA polymerase and 0.4 µM of the primers CamRpoB‐L and RpoB‐R (Primers listed in Table 1). After an initial denaturation step (95°C for 3 min), PCR involved 35 cycles of denaturation (94°C for 30 s), primer annealing (54°C for 30 s), chain extension (72°C for 30 s), and a final elongation step (72°C for 7 min). After gel electrophoresis in 3% agarose gel, PCR products were visualized with GR Green under UV light. The amplified DNA was purified by the GeneJET PCR Purification Kit (Thermo Fisher Scientific) and sequenced with CamRpoB‐L and RpoB‐R primers by Eurofins Genomics (Ebersberg). The sequences were analyzed using BioNumerics version 7.1 (Applied Maths) and standard Nucleotide BLAST (NCBI) and compared with the NCBI nucleotide collection database to verify the *Arcobacter* species.

## 3 RESULTS

**FIGURE 1:**
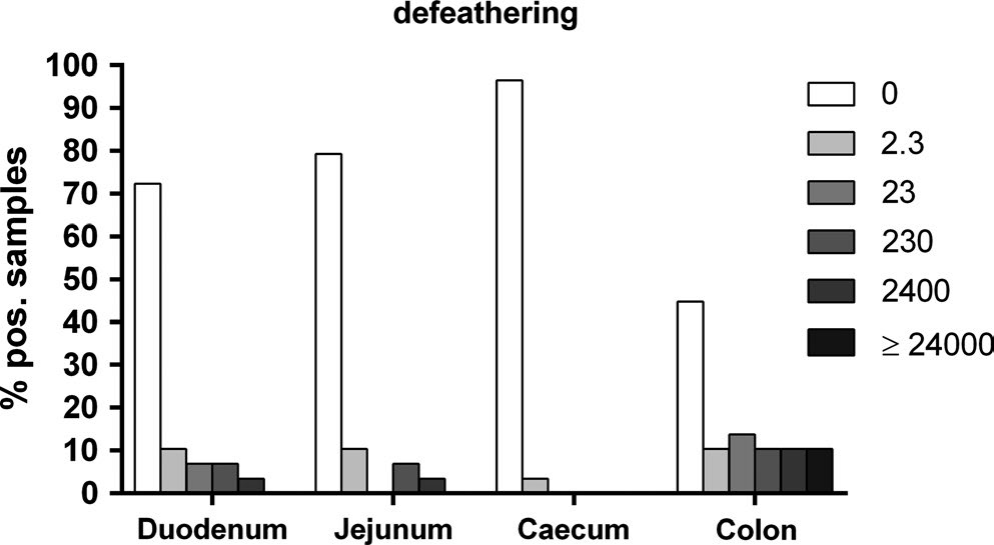
Percentages of *Arcobacter* spp. loads (MPN/g) in duodenal, jejunal, cecal, and colonic content after defeathering (*n* = 29)

**FIGURE 2:**
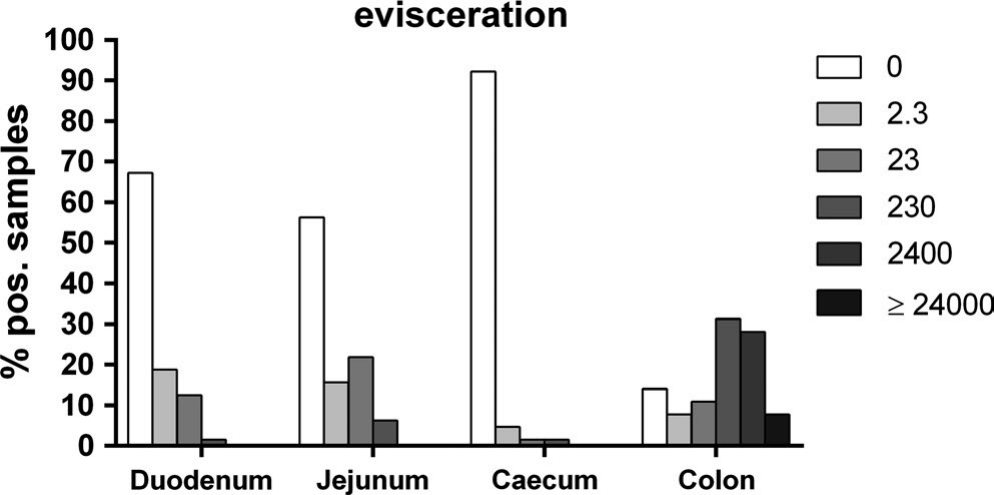
Percentages of *Arcobacter* spp. loads (MPN/g) in duodenal, jejunal, cecal, and colonic content after evisceration (*n* = 64)

*Arcobacter* spp. were detected in each of the 19 examined flocks at least at one of the four sampling sites. However, after the first two production steps, the prevalences of *Arcobacter* spp. in the four sections of the intestinal tract were low. After bleeding, *Arcobacter* spp. were only detected in the intestinal content of 6% (2/32) of the colonic samples whereas no *Arcobacter* spp. were detected in any intestinal content after scalding (Table 2). The *Arcobacter* load in both colonic content samples after bleeding was relatively low (2.3 and 230 MPN/g). In contrast, after defeathering and evisceration, the prevalences and *Arcobacter* loads increased. After defeathering, *Arcobacter* spp. were detected in the intestinal content of 28% (8/29) of the duodenal, 21% (6/29) of the jejunal, 3% (1/29) of the cecal, and 55% (16/29) of the colonic samples (Table 2). The highest *Arcobacter* load was determined in the colonic content (up to 24,000 MPN/g), followed by the duodenal and jejunal contents with up to 2,400 MPN/g, while in the single *Arcobacter*‐positive cecal sample a load of 2.3 MPN/g was determined (Figure 1). After evisceration, *Arcobacter* spp. were detected in the intestinal content of 33% (21/64) of the duodenal, 44% (28/64) of the jejunal, 8% (5/64) of the cecal, and 86% (55/64) of the colonic samples (Table 2). The highest *Arcobacter* load was determined in the colonic contents (up to 24,000 MPN/g), while in duodenal and jejunal contents up to 230 MPN/g were determined (Figure 2). One sample of cecal content was loaded with 230 MPN/g of *Arcobacter* spp., while the other four positive samples displayed lower loads (Figure 2).

**TABLE 2:** Prevalence of *Arcobacter* spp. in duodenal, jejunal, cecal, and colonic content of broiler chicken at four sampling sites in the slaughterhouse

Sampling site	% positive samples (positive/total)			
	Duodenum	Jejunum	Cecum	Colon
Bleeding	0% (0/32)	0% (0/32)	0% (0/32)	6% (2/32)
Scalding	0% (0/32)	0% (0/32)	0% (0/32)	0% (0/32)
Defeathering	28% (8/29)	21% (6/29)	3% (1/29)	55% (16/29)
Evisceration	33% (21/64)	44% (28/64)	8% (5/64)	86% (55/64)

Overall, in the majority of the 76 *Arcobacter*‐positive intestinal tracts detected so far, the highest *Arcobacter* load was detected in the colonic content, while the loads in the corresponding duodenal and jejunal contents were lower. Only in four intestinal tracts, the *Arcobacter* load was similar in the colonic and either duodenal or jejunal content. In one intestinal tract, the highest *Arcobacter* load was determined in the jejunal content, while in five intestinal tracts *Arcobacter* spp. were detected only in the duodenal or jejunal content but not in the colonic content.

**FIGURE 3:**
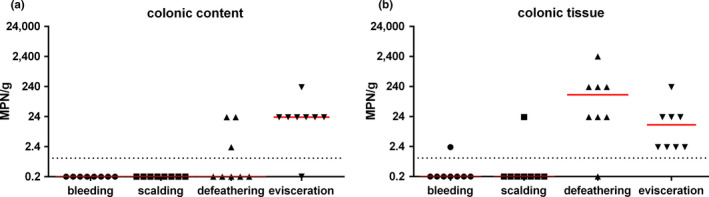
*Arcobacter* spp. loads (MPN/g) in colonic content (a) and colonic tissue (b) after bleeding, scalding, defeathering, and evisceration. Corresponding sections of eight carcasses were investigated at each sampling site

Additionally, eight samples of colonic content and the corresponding colonic tissue were examined after bleeding, scalding, defeathering, and evisceration. After bleeding and scalding, no *Arcobact*er spp. were detected in the colonic content, while one colonic tissue sample was tested positive for *Arcobacter* spp. after bleeding with a load of 2.3 MPN/g and one colonic tissue sample after scalding with a load of 23 MPN/g (Figure 3). After defeathering, *Arcobacter* spp. were detected in 38% (3/8) of the colonic content samples with loads up to 23 MPN/g, while *Arcobacter* spp. were determined in 88% (7/8) of the colonic tissue samples with a median load of 230 MPN/g. After evisceration, *Arcobacter* spp. were detected in 88% (7/8) of the colonic content samples and in all samples of colonic tissue with a median load of 23 MPN/g each (Figure 3).

Concerning scalding water and plucking finger samples, *Arcobacter* spp. were detected in 83% (5/6) of the scalding water samples with a median load of 2.3 MPN/ml. All six plucking fingers were contaminated with *Arcobacter* spp. with a median load of 24,000 MPN/ml.

Taken all intestinal samples together, *A*.* butzleri* was detected in 80% (74/93), *A*. *cryaerophilus* in 8% (7/93), and coinfections with both species were determined in 13% (12/93) of the *Arcobacter*‐positive intestinal tracts (49%, 93/189).

## 4 DISCUSSION

So far, the transmission route of *Arcobacter* spp. into the poultry, slaughterhouse, has not been clarified. Some authors suggest that *Arcobacter* spp. colonize the intestinal tract of chicken and thereby enter the slaughterhouse (Ho et al., 2008; Kabeya et al., 2003), while others were not able to detect *Arcobacter* spp. in the intestinal contents of broiler chicken (Atabay & Corry, 1997; Eifert, Castle, Pierson, Larsen, & Hackney, 2003; Houf et al., 2002). The present study tried to contribute to reveal the routes of transmission of *Arcobacter* spp. during processing in the chicken slaughterhouse. The intestinal contents of chicken carcasses were examined along the slaughter line, after bleeding, scalding, defeathering, and evisceration. However, by the methods applied, the three species *A*.* butzleri*,* A*.* cryaerophilus*, and *A*.* skirrowii* were mainly detected. Therefore, we cannot rule out whether other *Arcobacter* species were present or not.

The overall prevalence of *Arcobacter* spp. in intestinal tracts of broiler chicken determined in our study (49%) is in line with the prevalences determined in studies that detected *Arcobacter* spp. in the intestinal content of chicken (Ho et al., 2008; Van Driessche & Houf, 2007).

To reduce the impact of the flock on the prevalence at each sampling site, samples were collected at several processing points for each flock. Even though *Arcobacter* spp. were only detected sporadically in samples taken after bleeding and scalding, high loads and prevalences of *Arcobacter* spp. were detected in the intestinal contents and colonic tissues after defeathering and evisceration in each of the 19 flocks. In the cecal content, however, *Arcobacter* spp. were only sporadically detected at all four investigated processing steps. Therefore, the cecum is not likely to be the reservoir of *Arcobacter* spp. inside the chicken intestines, which has also been suggested by Ho et al. (2008). In the colonic section, the highest prevalences and bacterial loads were determined, which both declined toward the more orad situated duodenal and jejunal intestinal sections. After defeathering, higher prevalences and bacterial loads of *Arcobacter* spp. were determined in the colonic tissue compared with the colonic content. However, after the evisceration process, the prevalences and median bacterial loads did not differ between the colonic tissue and content. These data suggest that *Arcobacter* spp. are possibly localized in the tissue/mucus layer of the intestinal tract, as has already been reported for *Campylobacter* (Awad, Hess, & Hess, 2018). However, we cannot completely rule out that the *Arcobacter* spp. isolated from colonic tissue samples partially derives from residues of the colonic content. Further samples need to be examined to affirm colonic tissue/mucosa as an *Arcobacter* reservoir in chicken. Assuming *Arcobacter* spp. reside inside the chicken—but were not detected in our study in contents of the intestinal tracts and colonic tissues after bleeding and scalding—it can be speculated that certain processes (not yet determined) after scalding allowed detection of *Arcobacter* spp. at a later stage in the slaughter line (i.e., after defeathering). As no other major processing steps are between both sampling points and the time between both sampling stages is relatively short, the physical forces during the defeathering process should be considered in future investigations.

Furthermore, one has to consider possible cross‐contamination during the defeathering process. Several authors assumed that *Arcobacter* spp. can establish and proliferate in the environment inside the poultry slaughterhouse and several sources of *Arcobacter* contamination have been claimed, for example process water, slaughterhouse environment, and the defeathering machine itself (Ferreira, Fraqueza, Queiroz, Domingues, & Oleastro, 2013; Gude et al., 2005; Houf, De Zutter, Verbeke, Van Hoof, & Vandamme, 2003; Kjeldgaard, Jorgensen, & Ingmer, 2009). The few *Arcobacter*‐positive samples detected in the colonic content after bleeding and scalding with only low *Arcobacter* loads might rather indicate cross‐contamination of the colonic content originating during the transport to the slaughterhouse or the environment within (Corry & Atabay, 2001; Ho et al., 2008; Van Driessche & Houf, 2007). *Arcobacter* spp. are described as being able to survive the scalding temperatures, and therefore, the scalding water is suspected to contribute to the contamination within the slaughterhouse (Ho et al., 2008; Van Driessche & Houf, 2008). In our study, *Arcobacter* spp. were also detected in the scalding water samples. However, the *Arcobacter* loads determined in the scalding water (up to 230 MPN/ml) could not explain the higher *Arcobacter* loads in the intestinal contents determined after defeathering (up to 24,000 MPN/g). Therefore, an additional source with a consistent flow of high loads of *Arcobacter* spp. during the defeathering process, such as compartments of the defeathering machine (Plucker) or process water, h to be assumed.

Several characteristics of *Arcobacter* spp. might facilitate their ability to establish in the environment inside the slaughterhouse. Detection of *Arcobacter* in several water bodies has been reported (Hsu & Lee, 2015). Also, *Arcobacter* spp. can form biofilms under conditions most likely to be found in poultry slaughterhouses, and chicken meat juice supports the growth of *A*.* butzleri* at cold temperatures (Ferreira et al., 2013; Kjeldgaard et al., 2009). In combination with the ability to withstand certain disinfection substances that are generally used in slaughterhouses (Rasmussen, Kjeldgaard, Christensen, & Ingmer, 2013), *Arcobacter* spp. possibly reside and multiply in the environment inside the slaughterhouse. Contamination of plucking fingers by scalding water or contact with cross‐contaminated carcasses has been reported (Allen, Tinker, Hinton, & Wathes, 2003; Houf et al., 2003). In line with this, high loads of *Arcobacter* spp. (median of 24,000 MPN/ml) have been determined on the plucking fingers investigated in our study. Of all samples included within this study, the highest *Arcobacter* loads have been determined for plucking fingers. These data let us hypothesize that *Arcobacter* spp. can attach and possibly form biofilms on plucking fingers under the existing conditions. Therefore, the plucking fingers seem to be a potential source for contamination with *Arcobacter* spp. Assuming this process as sole cross‐contamination, the *Arcobacter* loads in the duodenal and jejunal contents need deeper consideration, since these orad parts of the intestines are more difficult to be cross‐contaminated as the more distal situated colon. It has to be analyzed whether the mechanical pressure released on the chicken carcasses during defeathering is as intense that reverse flow of *Arcobacter*‐contaminated material could be responsible for the detected loads of *Arcobacter* spp. in the duodenal and jejunal intestinal contents.

Taken together, further investigation is needed to clarify whether *Arcobacter* spp. in the intestinal contents of broilers derive solely from cross‐contamination during the defeathering process or if *Arcobacter* spp. also have a permanent reservoir inside the chicken intestinal tract.

## 5 CONCLUSION

In this study, we were not able to define the source of *Arcobacter* contamination in the poultry slaughterhouse. However, our data contributed to the understanding of *Arcobacter* transmission inside the poultry slaughterhouse and pointed out that cross‐contamination processes seem to be of multifactorial origin and the defeathering procedure might be of importance for *Arcobacter* cross‐contamination in the production line. Since *Arcobacter* spp. are commonly present on poultry products, the transmission routes of *Arcobacter* spp. into the poultry slaughterhouse need to be further investigated.

## CONFLICT OF INTERESTS

None declared.

## AUTHOR CONTRIBUTION

**Greta Goelz:** Conceptualization (lead); Formal analysis (equal); Writing‐original draft (equal); Writing‐review & editing (equal). **Antje Schoenknecht:** Conceptualization (supporting); Formal analysis (equal); Writing‐original draft (equal); Writing‐review & editing (equal). **Thomas Alter:** Conceptualization (lead); Funding acquisition (lead); Writing‐original draft (supporting); Writing‐review & editing (equal).

## ETHICS STATEMENT

None required.

## Data Availability

All data generated or analyzed during this study are included in this published article.
